# β-defensin 1 expression in HCV infected liver/liver cancer: an important role in protecting HCV progression and liver cancer development

**DOI:** 10.1038/s41598-017-13332-0

**Published:** 2017-10-17

**Authors:** Yue-Ming Ling, Jin-Yu Chen, Libin Guo, Chen-Yi Wang, Wen-Ting Tan, Qing Wen, Shu-Dong Zhang, Guo-Hong Deng, Yao Lin, Hang Fai Kwok

**Affiliations:** 1 Department of Infectious Diseases, Southwest Hospital, Third Military Medical University, Chongqing, 400038 P.R. China; 2 Department of Medical Laboratory, The 180th Hospital of PLA, Quanzhou, Fujian province 362000 P.R. China; 3 Department of Medical Quality Control, The 180th Hospital of PLA, Quanzhou, Fujian province 362000 P.R. China; 4 Faculty of Health Sciences, University of Macau, Avenida de Universidade, Taipa, Macau SAR P.R. China; 5 College of Life Sciences, Fujian Normal University, Fuzhou, Fujian Province P.R. China; 6 Centre for Cancer Research & Cell Biology (CCRCB) and Centre for Public Health (CPH), School of Medicine, Dentistry & Biomedical Sciences, Queen’s University Belfast, Belfast, UK; 7 Northern Ireland Centre for Stratified Medicine, Biomedical Sciences Research Institute, School of Biomedical Sciences, Ulster University, Londonderry, UK

## Abstract

β-defensin family plays a role in host defense against viral infection, however its role in HCV infection is still unknown. In this study, we demonstrated that β-defensin 1 was significantly reduced in HCV-infected liver specimens. Treatment with interferon and ribavirin upregulated β-defensin-1, but not other β-defensin tested, with the extent and duration of upregulation associated with treatment response. We investigated β-defensin family expression in liver cancer in publicly available datasets and found that among all the β-defensins tested, only β-defensin 1 was significantly downregulated, suggesting β-defensin 1 plays a crucial role in liver cancer development. Further analysis identified E-cadherin as the top positive correlated gene, while hepatocyte growth factor-regulated tyrosine kinase substrate as the top negative correlated gene. Expression of two proteoglycans were also positively correlated with that of β-defensin 1. We have also identified small molecules as potential therapeutic agents to reverse β-defensin 1-associated gene signature. Furthermore, the downregulation of β-defensin 1 and E-cadherin, and upregulation of hepatocyte growth factor-regulated tyrosine kinase substrate, were further confirmed in liver cancer and adjacent normal tissue collected from in-house Chinese liver cancer patients. Together, our results suggest β-defensin 1 plays an important role in protecting HCV progression and liver cancer development.

## Introduction

Hepatitis C virus (HCV) is a positive-stranded RNA virus with an estimated disease prevalence of 2–3% of the world’s population. HCV infection contributes to more than 70% of post-transfusion hepatitis, without proper treatment, HCV infection could lead to liver cirrhosis, liver failure and hepatocellular carcinoma^1^.

Defensin family is a group of small, disulphide-rich, cationic peptides, with highly diverse sequences and structures among eukaryotes. The family comprises three subfamilies (α, β and θ), mostly differ in length, their precursor structure, the location of the disulphide bonds between particular cysteines. α- and β-defensins are similar in their tertiary structure, which is difference from θ-defensin^2,3^.

All known β-defensin in mammals are encoded by DEFB genes that consist of 2 exons. There were 31 human β-defensin genes found, while β-defensin 1 (DEFB1) is recognized as the most important anti-microbial peptide in epithelial cells, with its action directed against both Gram-negative bacteria and fungal *Candidia* species. It has recently been found that β-defensin 1 prevents bacterial invasion for host defense by entrapping bacteria in extracellular net structures^4^. In addition, β-defensin 1 plays a role in innate immunity against viral infection, including HIV-1. A single nucleotide polymorphism in the promoter region of β-defensin 1 was associated with β-defensin 1 expression and the risk of HIV-1 infection^5^ while two other single nucleotide polymorphisms were shown to have protective role against HIV-1 infection and associate with mother-to-child transmission of HIV-1^6^. β-defensin 1 expression was found to be reduced in alveolar macrophages in HIV-infected patients^7^. Moreover, β-defensin 1 expression was induced upon infection of influenza, Herpes simplex and Sendai viruses and has been shown to protect host from influenza pathogenesis^8–11^.

Dysregulation of β-defensin 1 has also been demonstrated in various types of cancer. Decreased expression of β-defensin 1 was found in both renal and prostatic carcinoma^12^, suggesting its role as tumor suppressor in urological cancers probably through modulating apoptosis^13,14^. Moreover, decreased in β-defensin 1 expression was also found in oral squamous cell carcinoma^15,16^, while β-defensin 1 has been shown to suppress tumor migration and invasion, and demonstrated as an independent prognostic marker for oral squamous cell carcinoma^17^.

β-defensin 1 expression has been detected in liver in various mammalian species^18–20^. In human, β-defensin 1 expression in liver has been demonstrated to contribute to the biliary antimicrobial defense system^21^. However, the role of β-defensin 1 in HCV infection and carcinogenesis of the liver was largely unknown. In the present study, we investigated the expression of all the β-defensin genes in publicly available liver patient datasets, and have found that β-defensin 1 was the only β-defensin gene that were consistently downregulated in liver cancer compared to non-tumor liver specimens in 3 independent patient cohorts. β-defensin 1 expression was also decreased in HCV-infected patients, and that interferon treatment also modulated β-defensin 1 expression and this modulation may predict the response to interferon treatment. Further analyzing these patient cohorts resulted in identification of genes that are co-regulated with β-defensin 1 and small molecules that could reverse the cancer specific β-defensin 1-mediated gene signature.

## Results

### Downregulation of DEFB1 in HCV-infected liver specimens

**Figure 1:**
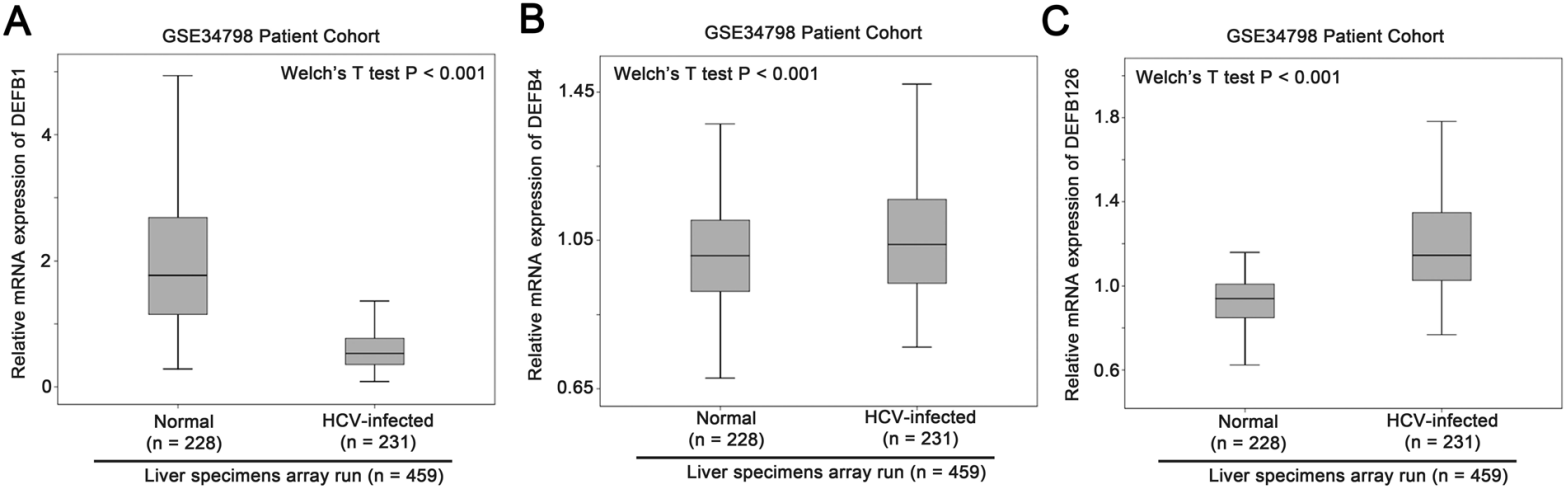
The expression of β-defensin 1 in normal and HCV infected liver. Box plots for mRNA expression of (**A**) β-defensin 1, (**B**) β-defensin 4 and (**C**) β-defensin 126 in normal and HCV-infected liver.

Since β-defensin has been shown to play important role in host defence against viral infection, we tested the expression level of β-defensin genes in a patient cohort consisting of more than 400 specimens from normal people and HCV patients following organ transplant. Only probesets for DEFB1, DEFB4 and DEFB126 were available in both HCV datasets and were analysed. In GSE34798, as shown in Fig. 1A, DEFB1 expression was downregulated in HCV-infected liver significantly compared to normal liver (P < 0.001). On the other hand, DEFB4 (Fig. 1B, P < 0.001) and DEFB126 (Fig. 1E, P < 0.001) mRNA expression levels were up-regulated.

### Treatment of interferon and ribavirin modulated DEFB1 expression in HCV-infected patients

**Figure 2:**
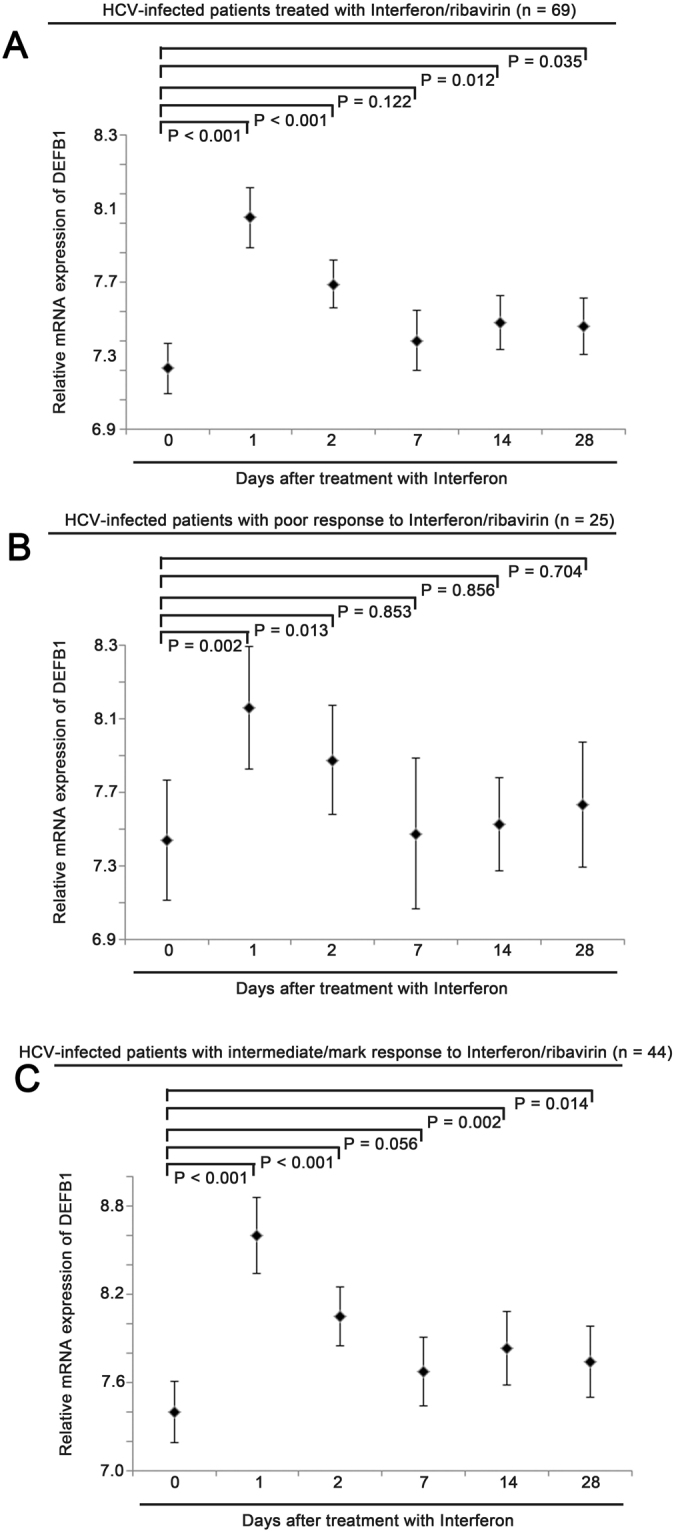
The expression of β-defensin 1 in peripheral blood mononuclear cells in patients treated with interferon and ribavirin. Error plots for mRNA expression of β-defensin 1 in peripheral blood mononuclear cells collected at different time point pre- or post-treatment with interferon and ribavirin in (**A**) all HCV infected patients, (**B**) HCV infected patients with poor response to treatment and (**C**) HCV infected patients with intermediate/mark response to treatment.

Since β-defensin genes were significantly modulated in HCV-infected liver, we went on to further analysed how this modulation may predict treatment response. In a patient cohort, GSE7123, consists of 397 specimens from 69 patients treated with interferon and ribavirin, with their blood samples collected at day 0, day 1, day 2, day 7, day 14 and day 28 post treatment available for microarray analysis. Only probesets for DEFB1, DEFB4 and DEFB126 were available for the analysis. We found that DEFB1 mRNA expression level was significantly upregulated at day 1, day 2, day 14 and day 28 post interferon and ribavirin treatment (Fig. 2A; P < 0.05). On the other hand, there was no significant change in DEFB4 expression level (Supplementary Figure 1A). For DEFB126, a significant downregulation of its mRNA was detected at day 1 and day 2 but not for those specimens collected at later time points (Supplementary Figure 1B).

### Upregulation of DEFB1 by interferon and ribavirin predicted the treatment response

As shown in Fig. 2B and C, upregulation of DEFB1 mRNA expression in response to treatment of interferon and ribavirin occurred in patients who either have poor or good response to the treatment. However, as shown in Fig. 2B, the upregulation of DEFB1 mRNA expression was less pronounced in patients who had poorer response to interferon and ribavirin, importantly, the upregulation was less sustainable as there was no statistically significant difference in DEFB1 mRNA expression at day 7 onwards post-treatment compared to pre-treatment level. In contrast, the upregulation of DEFB1 was more pronounced in patients with a better response to interferon and ribavirin (Fig. 2C). In addition, the upregulation of DEFB1 mRNA was significant even in day 14 and day 28 (Fig. 2C; P < 0.05), suggesting that higher level of and prolonged upregulation of DEFB1 detected in blood samples from HCV-infected patients may predict their response to treatment with interferon and ribavirin.

### Downregulation of DEFB1 in liver cancer

**Figure 3:**
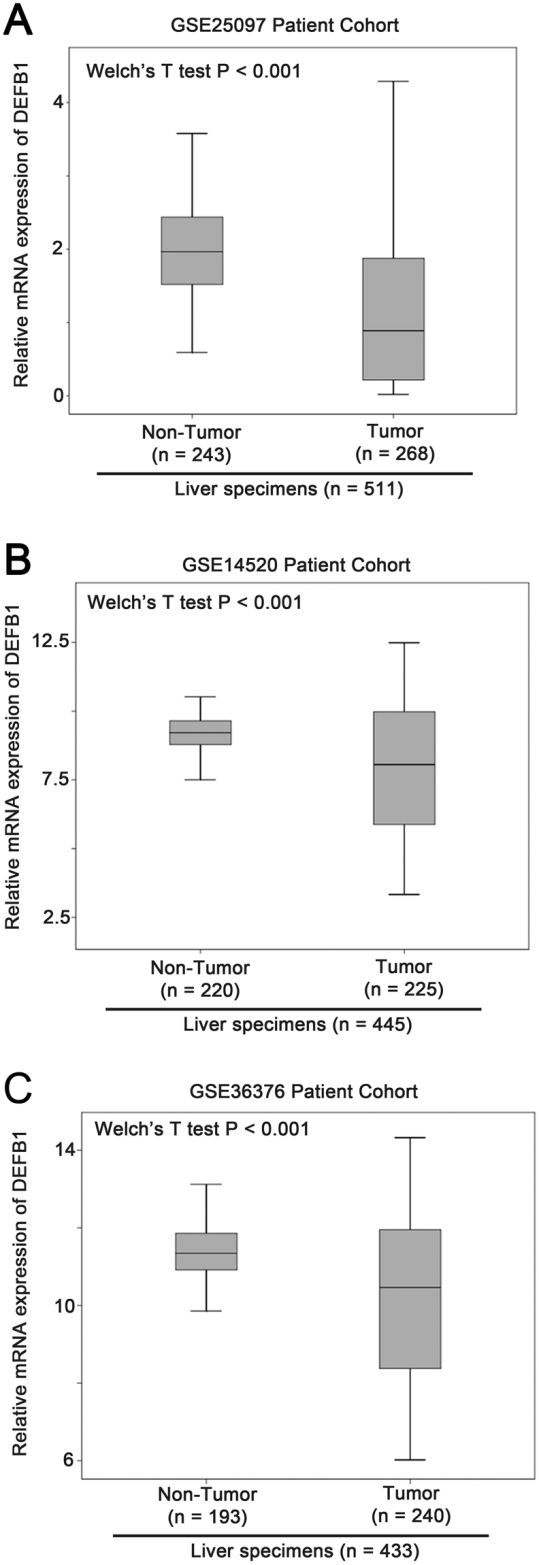
The expression of β-defensin 1 in non-tumor and tumor liver specimens. Box plots for mRNA expression of β-defensin 1 in non-tumor and tumor liver specimens in (**A**) GSE25097 (n = 511), (**B**) GSE14520 (n = 445) and (**C**) GSE36376 (n = 433).

All the probesets for β-defensin family available in the 3 cohorts (GSE25097, GSE14520 and GSE36376) with more than 400 specimens consisting at least 200 samples of liver cancer in a single platform were extracted and analysed. The expression levels of β-defensin genes were compared between non-tumor and tumor specimens. As shown in Supplementary Tables 1A–C, we found that only β-defensin 1, DEFB1, expression was consistently and significantly downregulated in liver cancer specimens compared to non-tumor specimens, but not that of other β-defensin genes. As shown in Fig. 3A–C, the downregulation of β-defensin 1 in liver specimens was statistically significant in GSE25097, GSE14520 and GSE36376 cohorts (P < 0.001).

### Identification of DEFB1 co-regulated genes in liver cancer patient cohorts

To investigate the mechanisms related to the downregulation of DEFB1 in liver cancer, we further analysed the 3 liver cancer patient cohorts to identify genes that had strong correlation with DEFB1 in terms of their mRNA expression. Specimens in these 3 cohorts were stratified based on the median expression level of DEFB1 mRNA as cut-off and genes that are differentially expressed were identified. Among the top 18 genes, CDH1 gene encoding E-cadherin, which has been shown to suppress liver carcinogenesis^22^, was listed at the top with positive correlation based on the lowest p-values among the 3 cohorts, while HGS gene encoding hepatocyte growth factor-regulated tyrosine kinase substrate (HGS), which has been shown to be required for liver cancer cell survival with high level of β-catenin signalling^23^, was listed at the top, while HGS was listed at the top with negative correlation based on the lowest p-values among the 3 cohorts. We also found two proteoglycan genes, DCN gene encoding Decorin, which has been shown to prevent hepatocarcinogenesis^24^, and LUM gene encoding Lumican, which has been shown to be required for hepatic fibrosis^25^, among these 18 genes. These few genes were further analysed.

**Figure 4:**
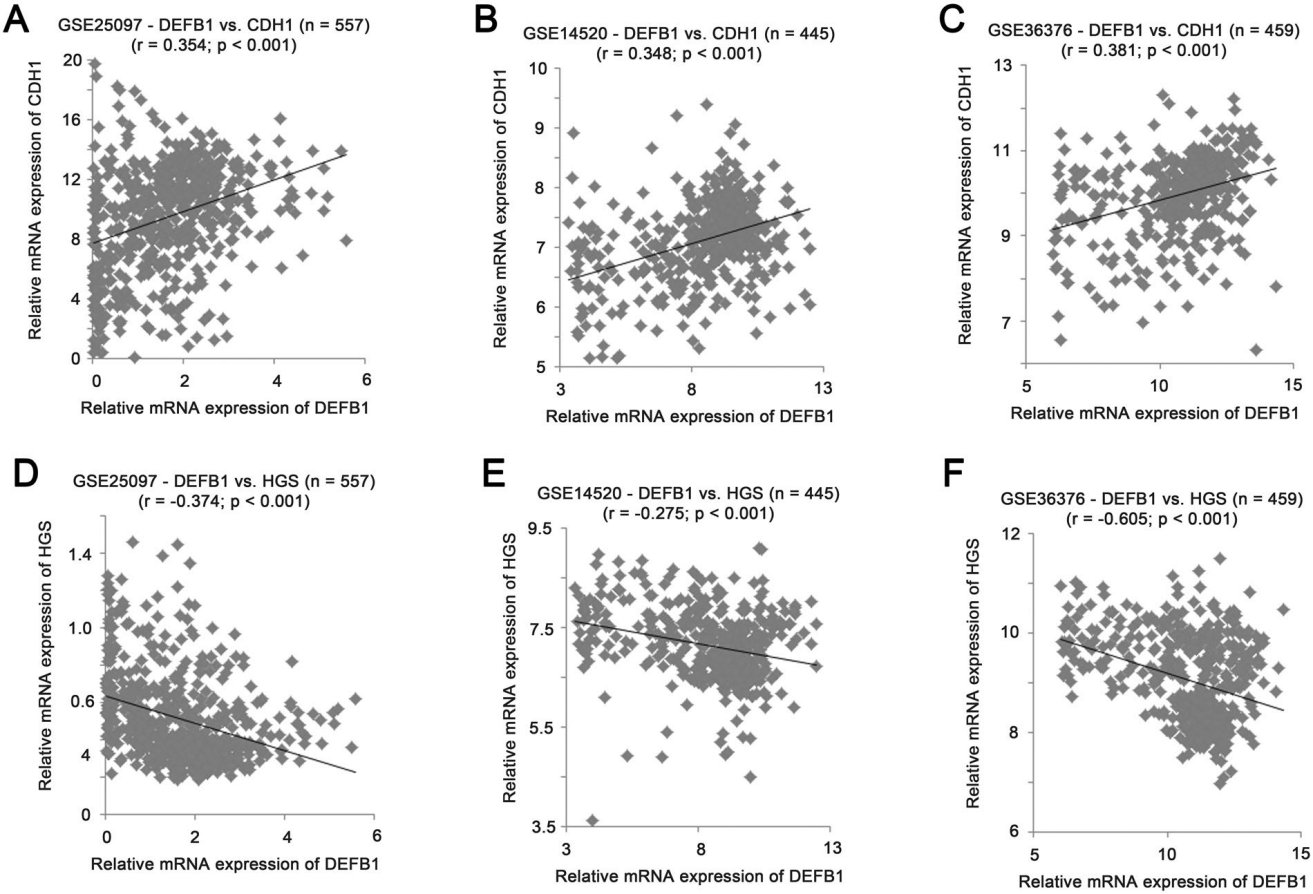
The correlation of mRNA expression levels of β-defensin 1 and CDH1, and β-defensin 1 and HGS. Scatter plots for mRNA expression levels of DEFB1 and CDH1 in liver specimens in (**A**) GSE25097 (n = 557), (**B**) GSE14520 (n = 445) and (**C**) GSE36376 (n = 459). Scatter plots for mRNA expression levels of DEFB1 and HGS in liver specimens in (**D**) GSE25097 (n = 557), (**E**) GSE14520 (n = 445) and (**F**) GSE36376 (n = 459).

As shown in Fig. 4, the mRNA expression levels of DEFB1 and CDH1 were positively correlated in GSE25097 (Fig. 4A; r = 0.354, P < 0.001), GSE14520 (Fig. 4B; r = 0.348, P < 0.001) and GSE36376 (Fig. 4C; r = 0.381, P < 0.001) liver cancer patient cohorts. On the other hand, the mRNA expression levels of DEFB1 and HGS were negatively correlated in GSE25097 (Fig. 4D; r = −0.374, P < 0.001), GSE14520 (Fig. 4E; r = −0.275, P < 0.001) and GSE36376 (Fig. 4F; r = −0.605, P < 0.001).

**Figure 5:**
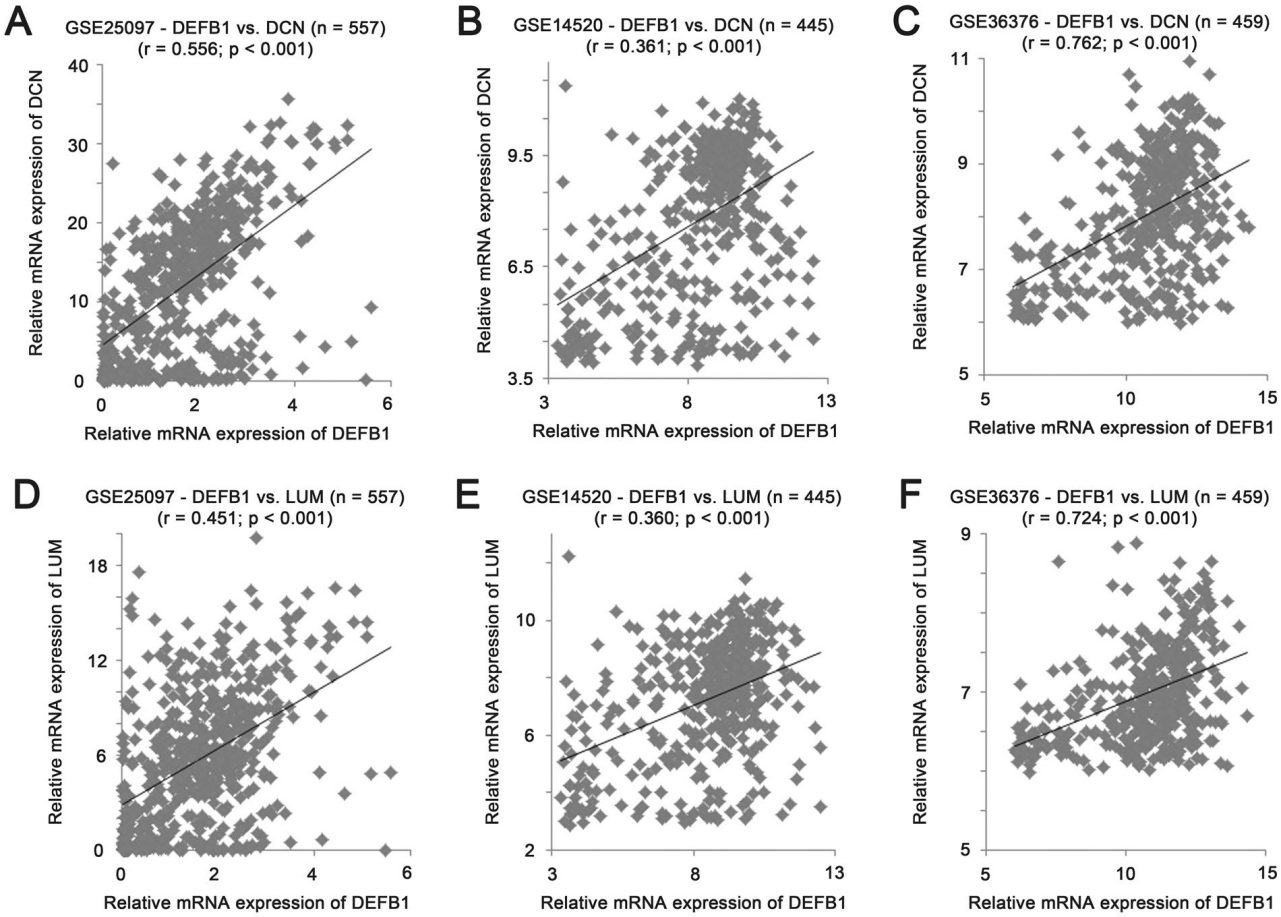
The correlation of mRNA expression levels of β-defensin 1 and DCN, and β-defensin 1 and LUM. Scatter plots for mRNA expression levels of DEFB1 and DCN in liver specimens in (**A**) GSE25097 (n = 557), (**B**) GSE14520 (n = 445) and (**C**) GSE36376 (n = 459). Scatter plots for mRNA expression levels of DEFB1 and LUM in liver specimens in (**D**) GSE25097 (n = 557), (**E**) GSE14520 (n = 445) and (**F**) GSE36376 (n = 459).

For the two proteoglycan genes, the mRNA expression levels were both positively correlated with that of DEFB1. The mRNA expression levels of Decorin (DCN) and DEFB1 were positive in GSE25097 (Fig. 5A; r = 0.556, P < 0.001), GSE14520 (Fig. 5B; r = 0.361, P < 0.001) and GSE36376 (Fig. 5C; r = 0.762, P < 0.001). Similarly, the mRNA expression levels of Lumican (LUM) and DEFB1 were positively correlated in GSE25097 (Fig. 5D; r = 0.451, P < 0.001), GSE14520 (Fig. 5E; r = 0.360, P < 0.001) and GSE36376 (Fig. 5F; r = 0.724, P < 0.001).

### Downregulation of CDH1, DCN and LUM, and upregulation of HGS in liver cancer

**Figure 6:**
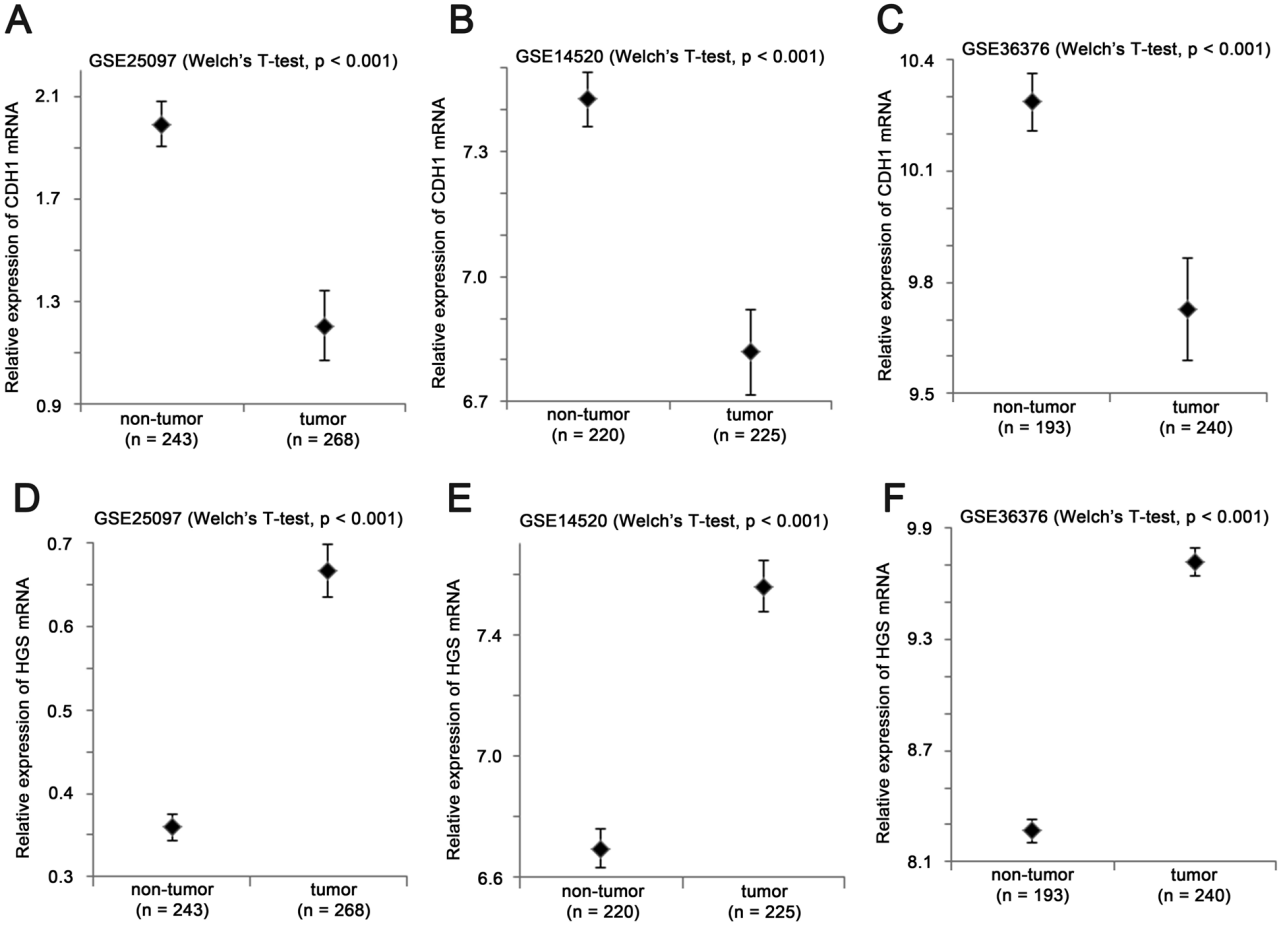
The expression of CDH1 and HGS in non-tumor and tumor liver specimens. Box plots for mRNA expression of CDH1 in non-tumor and tumor liver specimens in (**A**) GSE25097 (n = 511), (**B**) GSE14520 (n = 445) and (**C**) GSE36376 (n = 433). Box plots for mRNA expression of HGS in non-tumor and tumor liver specimens in (**D**) GSE25097 (n = 511), (**E**) GSE14520 (n = 445) and (**F**) GSE36376 (n = 433).

We further investigated whether expression levels of CDH1 and HGS were different between non-tumor and tumor specimens. We found that CDH1 expression was significantly and consistently downregulated in liver cancer specimens compared to non-tumor specimens in GSE25097 (Fig. 6A; P < 0.001), GSE14520 (Fig. 6B; P < 0.001) and GSE36376 (Fig. 6C; P < 0.001), while HGS expression was significantly and consistently upregulated in liver cancer specimens compared to non-tumor specimens in GSE25097 (Fig. 6D; P < 0.001), GSE14520 (Fig. 6E; P < 0.001) and GSE36376 (Fig. 6F; P < 0.001).

**Figure 7:**
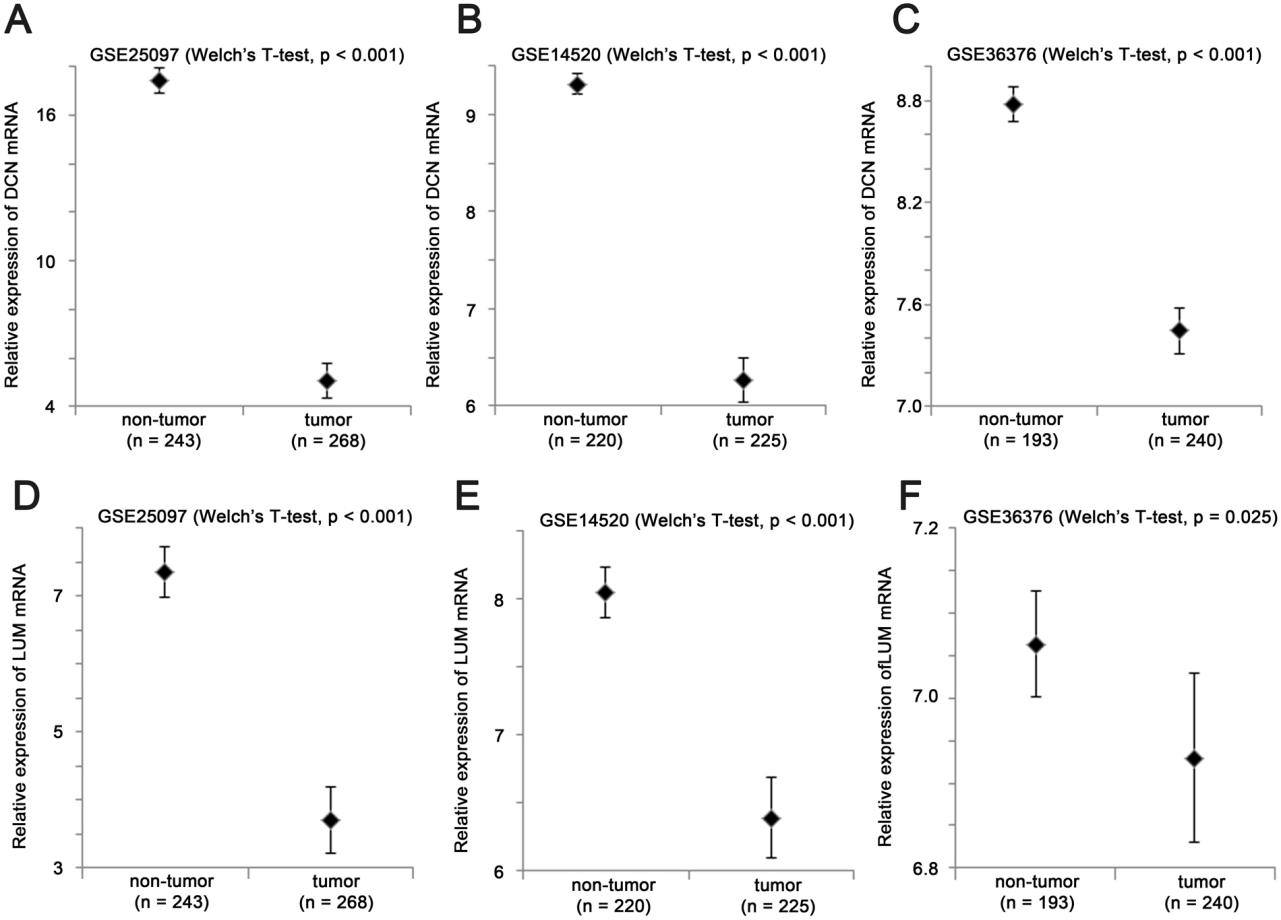
The expression of DCN and LUM in non-tumor and tumor liver specimens. Box plots for mRNA expression of DCN in non-tumor and tumor liver specimens in **(A**) GSE25097 (n = 511), (**B**) GSE14520 (n = 445) and (**C**) GSE36376 (n = 433). Box plots for mRNA expression of LUM in non-tumor and tumor liver specimens in (**D**) GSE25097 (n = 511), (**E**) GSE14520 (n = 445) and (**F**) GSE36376 (n = 433).

On the other hand, the two proteoglycan genes, DCN and LUM, were both downregulated in liver cancer. As shown in Fig. 7, liver cancer specimens had a significantly lower level of DCN in GSE25097 (Fig. 7A; P < 0.001), GSE14520 (Fig. 7B, P < 0.001) and GSE36376 (Fig. 7C, P < 0.001) compared to non-tumor specimens. Similar LUM expression level was lower in liver cancer specimens compared to non-tumor specimens in GSE25097 (Fig. 7D; P < 0.001), GSE14520 (Fig. 7E, P < 0.001) and GSE36376 (Fig. 7F, P = 0.025).

### Identification of small molecules that could reverse the DEFB1 co-regulated gene signature

**Figure 8:**
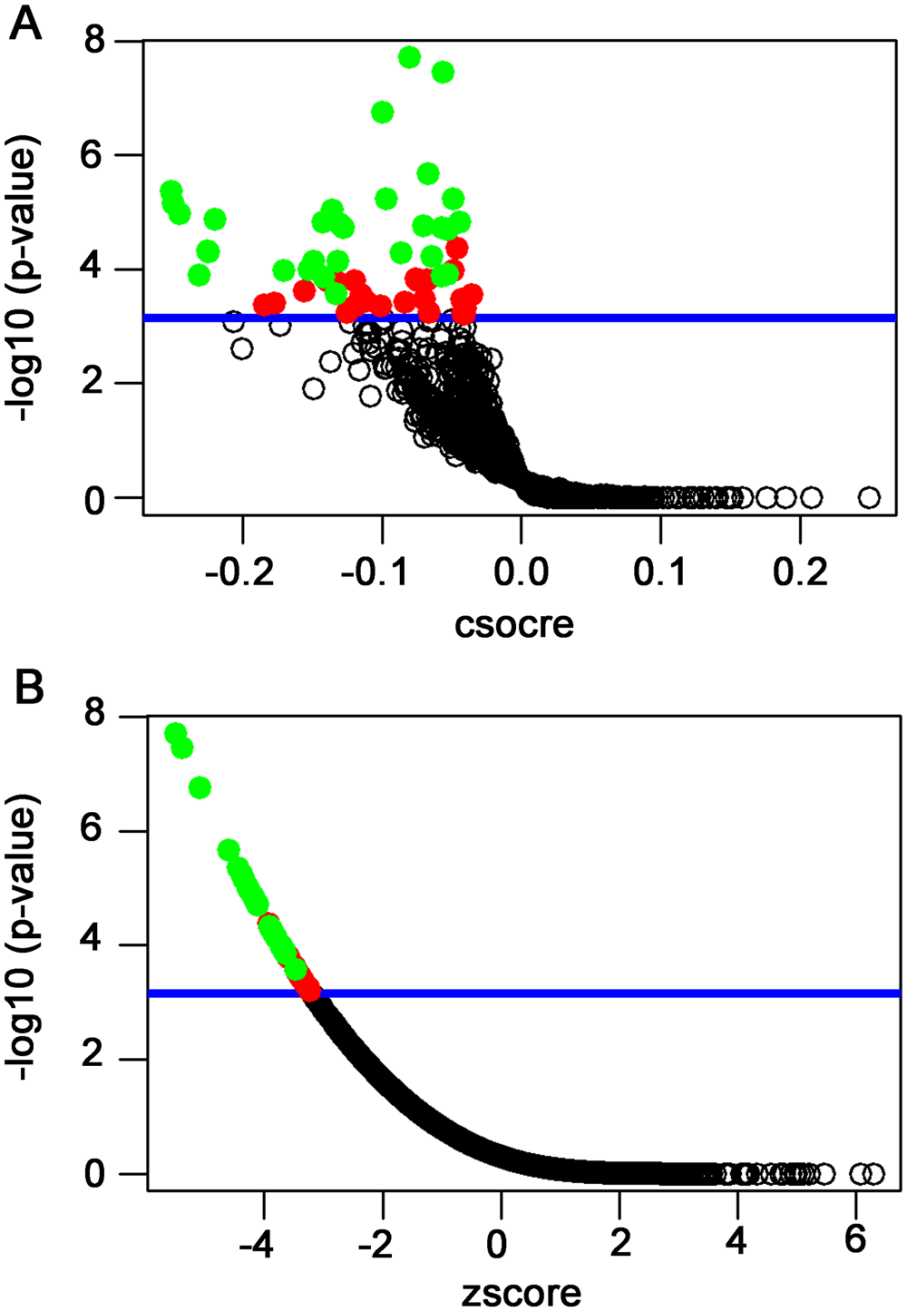
The results of connectivity mapping with the DEFB1 gene signature. (**A**) The raw connection scores (cscore). (**B**) The normalized connection scores (zscore). Each data point on these graphs represents a compound screened. The blue line indicates the threshold of statistical significance. Those data points above the blue line have significant connectivity mapping scores with the DEFB1 gene signature (significance mark = 1). Show in green are the significant drugs (listed in Supplementary Table S2) that are also highly stable under gene signature perturbations (perturbation stability = 1).

Among the top 18 probesets identified based on DEFB1 expression, we found that 16 of them, including CDH1, COMT, DCN, LUM, HSD17B2, UGP2, CYFIP2, AFM, TPD52L1, HPX, STARD5, EPHA3, SRPX, HGS, PDGFRA, RRAGD and FMO3, were consistently differentially expressed in liver cancer and non-tumor specimens. Thee 16 genes together with DEFB1 genes were used as a DEFB1 gene signature for identification of small molecule that could reserve the expression of these genes in connectivity mapping. As shown in Fig. 8 and Supplementary Table S2, among small molecules with a significant connectivity mapping score of 1 and a perturbation stability of 1, the top three small molecules were articaine, selegiline and ribavirin, several anthracyclines were also identified, including idarubicin, daunorubicin and doxorubicin, while erlotinib, which has been tested in liver cancer in clinical trial was also identified.

### Expression of β-defensin 1 E-cadherin and HGS in human liver specimens

**Figure 9:**
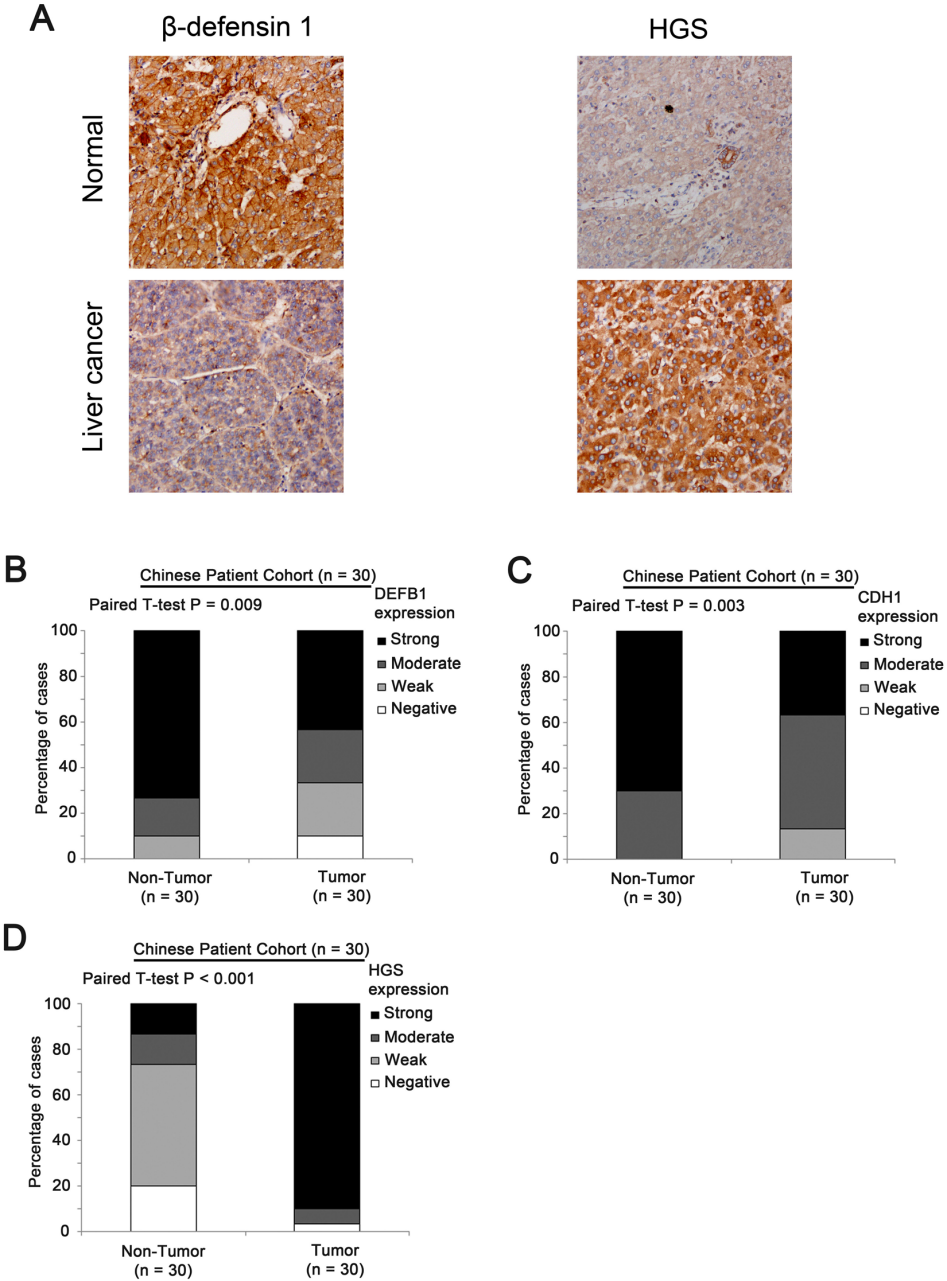
The results of immunohistochemical staining. (**A**) Representative images of immunohistochemical staining of β-defensin 1, E-cadherin and HGS in both cancerous and normal liver specimens. (**B**–**D**) The percentage of cases stained with different levels of **(B**) β-defensin 1, (**C**) E-cadherin and (**D**) HGS.

To confirm our finding in publicly available liver cancer patient datasets using mRNA expression, a Chinese liver cancer patient cohort with 30 patients with available liver cancer and paired adjacent normal liver specimens were included in the present study, and the protein expression of these genes were analysed by immunohistochemical staining, and we found that, similar to the findings in publicly available datasets, protein expression of β-defensin 1 (Fig. 9A and B; P = 0.009) and E-cadherin (Fig. 9A and C; P = 0.003) was downregulated while that of HGS was upregulated (Fig. 9A and D; P < 0.001) in liver cancer specimens compared to adjacent normal liver specimens.

## Discussion

In the present study, we have shown that DEFB1 was downregulated in HCV-infected specimens, while it was upregulated by treatment of interferon and ribavirin, and the degree and duration of upregulation of DEFB1 predicted the response to treatment. DEFB1 was also downregulated in liver cancer specimens compared to non-tumor specimens. Identification of co-regulated genes has shown that DEFB1 was positively correlated with CDH1, and two proteoglycan genes, DCN and LUM, and negatively correlated with HGS. These genes were differentially expressed in liver cancer and non-tumor liver specimens too. By using connectivity mapping, we have shown few chemicals, including ribavirin, anthracyclines and erlotinib, which were potential therapeutic agents for treating liver cancer, especially those with DEFB1 downregulated.

α-defensin was shown to be increased in peripheral blood mononuclear cells in patients with HCV infection^26^, on the other hand, β-defensin 3 has been shown to be upregulated by HBV and this upregulation actually prevented intrauterine transmission of HBV to infants^27^. While β-defensin 1 has been shown to be upregulated in cirrhotic liver, its expression in HCV-infected liver was still unknown^28^. In the present study, we found that the expression of β-defensin 1 was significantly reduced in HCV-infected liver compared to normal liver, and importantly, treatment with interferon and ribavirin resulted in upregulation of β-defensin 1 and the higher extent and longer duration of this upregulation was associated with a better response to the treatment. Our results suggest that β-defensin 1 expression may play a protective role in HCV progression and that expression of β-defensin 1 may be a potential predictive marker for treatment efficacy of interferon and ribavirin. Interferon and ribavirin was the standard of care for treatment of HCV prior to the discovery of direct anti-viral agents (DAA)^29^. Although this regimen has largely been replaced by DAA such as Sofosbuvir^30^ and Daclatasvir^31^, many countries still rely on interferon and ribavirin as the first line agent for HCV infected patients, and as a results, prediction of response to interferon and ribavirin may identify patients who need to be monitor closely and considered to switch to DAA earlier. Prospective clinical trial may be required to further confirm this retrospective findings.

To the best of our knowledge, the role of β-defensin 1 in liver cancer has not been established. The present report is the first one to demonstrate β-defensin 1, but not other β-defensins, were significantly downregulated in liver cancer specimens compared to non-tumor liver specimens consistently in 3 independent liver datasets with more than 400 specimens in each of them, suggesting β-defensin 1 may play a protective role in hepatic carcinogenesis. Although the mechanism has not been fully elucidated, the identification of CDH1, E-cadherin, as one of the top co-regulated genes with β-defensin 1 consistently in 3 independent liver dataset has shed light on the role of β-defensin 1 in liver cancer. E-cadherin has been shown to play an important role in liver cancer including early hepatocellular carcinoma recurrence after surgery^32^, enhancing cell invasion and migration^33^, promoting intrahepatic metastasis^34^ and extrahepatic metastasis^35^, and its expression has also been demonstrated as a potential prognostic marker^36–38^. Further *in vitro* analysis is required to investigate how β-defensin 1 and E-cadherin interact and how this interaction contributes to hepatocellular carcinoma. On the other hand, expression of HGS, hepatocyte growth factor-regulated tyrosine kinase substrate was found to be the negatively correlated with β-defensin 1 consistently in the 3 independent datasets. As HGS expression has been shown to be required for liver cancer cell survival in the presence of β-catenin signaling^23^, future study may focus on how β-defensin 1 may contribute to the synthetic lethal interaction between β-catenin and HGS in liver cancer. Importantly, the findings for mRNA in publicly available datasets could be further confirmed in our in-house Chinese liver cancer patient cohort consisting of 30 pairs of liver cancer and adjacent normal liver specimens, where protein expression of β-catenin, E-cadherin and HGS has been examined. Our protein data further confirm that β-catenin and E-cadherin may be a tumor suppressor, while HGS may be an oncogene in liver cancer.

Proteoglycan plays a key role in liver fibrogenesis; its synthesis is activated during liver injury^39^. Its role in liver cancer has also recently been revealed^40^. Most importantly, they interact with regulatory molecules and signaling pathways through their GAG chains to modulate the activities of these pathways, including growth factors, cytokines and hormonal pathways. The composition of proteoglycans would change during liver cancer development, suggesting their potential role as prognostic marker and therapeutic target^40^. In the present study, we found that the expression levels of two proteoglycans, decorin and lumican, were both significantly positively associated with β-defensin 1 expression, and reduced in liver cancer. Although the role of lumican in liver cancer is still unknown, decorin has been shown to play a protective role in liver cancer^40^, in line with our results that decorin was significantly reduced in liver cancer specimens. Decorin exerts its tumor suppressive role in liver cancer may attribute to its ability to interact multiple signaling pathway, including downregulation of β-catenin and Myc expression^41^, and activation of p21 and p57 with tumor suppressive functions^42,43^. As β-catenin activity was modulated by decorin while HGS has been shown to be required for survival in β-catenin activated background, the role of β-defensin 1 plays in β-catenin-mediated liver cancer development with this decorin- β-catenin-HGS axis warrant further confirmation and investigation.

By connectivity mapping, we have identified several small molecules that could reverse β-defensin 1-gene signature. Among these, the top two small molecules, articaine and selegiline, have not been investigated in details in hepatocellular carcinoma. However, ribavirin was extensively studied and was shown to play a protective role in liver cancer development, especially for patients with chronic hepatitis C infection^44,45^. Several anthracyclines have also been identified by connectivity mapping. Anthracyclines are one of the most active agents against hepatocellular carcinoma^46,47^, while defensins have been shown to alter membrane permeability and thereby the cytotoxic activity of doxorubicin against multidrug-resistant tumor cells^48^. Further study may investigate on whether β-defensin 1 expression may alter the sensitivity of hepatocellular carcinoma towards anthracyclines. EGFR signaling has been shown to play an important role in liver diseases^49^ while Erlotinib, an EGFR inhibitor, was suggested to be an potential therapeutic agent^50^ until the failure to demonstrate its efficacy in a phase III trial^51^.Although study on the combination of bevacizumab, an anti VEGFa antibody, and erlotinib has yielded promising results^52^, the clinical use of erlotinib in hepatocellular carcinoma has yet to be elucidated, especially, whether expression of β-defensin 1 could be a predictive biomarker for erlotinib warrant further investigation.

In conclusion, our study is the first to demonstrate that β-defensin 1 was reduced in HCV-infected patients while was upregulated upon interferon and ribavirin treatment with the extent and duration of upregulation associated with treatment response. In addition, our study is also the first to show that β-defensin 1 was downregulated in liver cancer with several potential interacting genes and therapeutic agents against β-defensin 1 being identified and discussed.

## Materials and Methods

### Extraction of clinical and microarray gene expression data from hepatocellular carcinoma and HCV-infected liver dataset

Three datasets with more than 400 samples in a single microarray platform tested for gene expressions in hepatocellular carcinoma were identified; including GSE14250^53^, GSE25097^54^, and GSE36376^55^. Two datasets with more than 300 samples in a single microarray platform tested for gene expressions in HCV-infected liver were also identified; GSE34798^56^ and GSE7123^57^. The data was extracted and analysed as previously described^58^. Briefly, the GEO website has standardized URLs for its individual data sets (DataSeries as referred to on GEO), and for each GEO DataSeries, links are provided to the Series Matrix File(s) which contain the expression values for each gene (probeset) and each sample. Microarray gene expression data were retrieved from the data matrices deposited to GEO by the original authors. Data normalization methods were specified under each DataSeries on GEO. Specifically, the RMA (Robust Multi-array Average) method was used to obtain probe set expression summaries for GSE14520 and GSE25097, and the quantile normalization method was used to obtain expression value for dataset GSE36376. For GSE34798, data was normalized within arrays with the loess method, and then normalized between arrays with the quantile normalization method and log2 transformed. For GSE7123, the average intensity on each array was normalized by global scaling to a target intensity of 1000; Data were then processed using the Affymetrix Microarray Suite 5 (MAS5) algorithm. All these were commonly used and widely accepted microarray gene expression data normalization methods as adopted by the original authors. Once the gene expression matrices were successfully obtained, R scripting was used to extract the expression values of a small number of genes of interest and also the clinical data available from the GEO data matrixes. All the β-defensin genes were extracted if probes were available for detecting their expression in the microarray platform. The information of included datasets is listed in Table 1.

**Table 1:** Information of the datasets included in the study.

Dataset	No. of Sample	Description
GSE34798	459	111 liver biopsy specimens collected longitudinally from 57 HCV-infected liver transplant patients
GSE7123	397	Consisting of 69 patients with gene expression arrays before and during treatment of HCV with interferon and ribavirin with time series of day 0, 1, 2, 7, 14 and 28
GSE25097	557	Consisting of 268 liver tumor, 243 adjacent non-tumor, 40 cirrhotic and 6 healthy liver specimens
GSE14520	488	Two microarray platforms; only specimens in GPL3921 platform was included totalling of 445 specimens, of which 220 was non-tumor and 225 was tumor liver specimens
GSE36376	433	Consisting of 193 adjacent non-tumor and 240 tumor liver specimens with gene profiling data

### Identification of β-defensin 1 co-expressing genes

Median expression of β-defensin 1 gene was used as a cut-off to stratify the specimens into the high and low expression subgroups. The gene expression patterns of patients in β-defensin 1-low subgroup and those in the β-defensin 1-high subgroup were compared. Probesets that were differentially expressed between these two subgroups were identified by 2-sample Welch’s T-test. This test was used to avoid the type I error due to unequal variances of the values of probesets between subgroups. Briefly, a Welch’s t test was applied to each probeset corresponding to a certain gene in the data matrix using our own Java application MyStats. *P* values and the differential expression in fold changes for all the probesets were generated as tab-delimited worksheets of Excel for further analysis. The genes were ranked based on the *p* value for all the probesets available for a particular gene and the top 18 genes were extracted from the GEO database for further analysis.

### Identification of potential therapeutic small molecule downregulated β-defensin 1 and its co-regulated genes through connectivity mapping

Gene expression connectivity mapping was performed using Statistically Significant Connection’s Map (sscMap) to identify candidate small molecule compounds that may reverse the reduced expression of β-defensin 1 gene and its associated gene expression signature^59–61^. The relevant probes were input to the Java application sscMap^61^ as a query signature, and its association with the 6000 gene expression profiles generated by treating cancer cells with over 1000 small molecules were compared. The gene signature perturbation procedure, which increases the specificity of the output results, was applied as previously described^62^. All the small molecular compounds that were negatively associated with the input were sorted and ranked by their *p*-value, perturbation stability and standardized connection score. The *p*-value that was considered significant was set at a stringent threshold (p = 1/1309), ensuring that the results generated by sccMap would yield a maximum of one false positive small molecule over the 1309 small molecules tested in the sccMap^62^.

### Patients and specimens of the Chinese liver cancer patient cohort

The study cohort was composed of samples from 30 patients (17 men and 13 women, mean age: 59.5 years) with liver cancer, who had a liver resection at The 180^th^ Hospital of PLA (Quanzhou, Fujian) between January 2016 and June 2017. Following surgery, routine chemotherapy was given to patients with advanced disease and no radiation treatment was administered to any of the patients. Eligibility criteria for patients included in this study were: (1) histologically proven liver cancer; (2) no history of other malignancy tumor; (3) no prior neoadjuvant chemotherapy. All methods were performed in accordance with approved institutional guidelines, and experimental protocols were approved by the Research Ethics Committee of The 180^th^ Hospital of PLA, Quanzhou, Fujian. Samples were collected in accordance with international standards for research ethics and were approved by the local institutional review board (The 180^th^ Hospital of PLA, Quanzhou, Fujian), with all patients giving informed consent.

### Immunohistochemical staining

Paraffin blocks that contained sufficient formalin-fixed specimens were serial sectioned at 4μm and mounted on silane-coated slides for immunohistochemistry analysis. The sections were deparaffinized with dimethylbenzene and rehydrated through 100, 100, 95, 85, and 75% ethanol. Antigen retrieval treatment was done in 0.01 mol/L sodium citrate buffer (autoclaved at 121 °C for 2 mins, pH 6.0) and endogenous peroxidase was blocked by incubation in 3% H_2_O_2_ for 10 mins at room temperature. The sections were then washed in PBS and blocked with 10% goat serum (ZhongShan Biotechnology, China) for 30 mins and incubated with rabbit anti-human CDH1 (20874-1-AP, 1:200 dilution, proteintech, polyclonal) or HGS (10390-1-AP, 1:250 dilution, proteintech, polyclonal) antibody or rabbit anti-human DEFB1 (14738-1-AP, 1:100 dilution, proteintech, polyclonal) in a humidified chamber at 4 °C overnight. Following three additional washes in PBS, the sections were incubated with HRP-conjugated secondary antibody for 30 mins at room temperature. The visualization signal was developed with diaminobenzidine (DAB) solution and all slides were counterstained with 20% hematoxylin. Finally all slides were dehydrated and mounted on cover slips. For negative controls, the primary antibody diluent was used to replace primary antibody.

### Evaluation of immunohistochemical staining

The IHC-stained tissue sections were reviewed under microscope by 2 pathologists who were blinded to the clinical parameters, and scored independently according to the intensity of cellular staining and the proportion of stained tumor cells. The staining intensity was scored as 0 (no staining), 1 (weak staining, light yellow), 2 (moderate staining, yellow brown), and 3 (strong staining, brown).

### Statistical analysis

All statistical analyses were performed using SPSS19.0. Welch’s T test was used to compare gene expression in different types of specimens. Paired T test was used to compare gene expression in the same patients at different time points. Spearman’s Rank test was used to test the correlation between expression of two genes.

## Electronic supplementary material

Supplementary Figure 1, Supplementary Table 1 and 2
