# Supplementary material for: β-defensin 1 expression in HCV infected liver/liver cancer: an important role in protecting HCV progression and liver cancer development
Source: Sci Rep. 2017 Oct 17;7:13404. doi: 10.1038/s41598-017-13332-0 (PMC5645372; doi:10.1038/s41598-017-13332-0)
Supplement: Supplementary file 1 — Supplementary Figure 1, Supplementary Table 1 and 2 [file 41598_2017_13332_MOESM1_ESM.pdf]

β-defensin 1 expression in HCV infected liver/liver cancer: an important role in protecting HCV progression and liver cancer development

Yue-Ming Ling[1,2], Jin-Yu Chen[3], Libin Guo[4], Chen-Yi Wang[5], Wen-Ting Tan[1], Qing Wen[6], Shu-Dong Zhang[7], Yao Lin[5]*, Hang Fai Kwok[4]*, Guo-Hong Deng[1]*

[1]Department of Infectious Diseases, Southwest Hospital, Third Military Medical University, Chongqing 400038, P.R. China

[2]Department of Medical Laboratory, The 180th Hospital of PLA, Quanzhou, Fujian province, 362000, P.R. China

[3]Department of Medical Quality Control, The 180th Hospital of PLA, Quanzhou, Fujian province, 362000, P.R. China

[4]Faculty of Health Sciences, University of Macau, Avenida de Universidade, Taipa, Macau SAR

[5]College of Life Sciences, Fujian Normal University, Fuzhou, Fujian Province, P.R. China

[6]Centre for Cancer Research & Cell Biology (CCRCB) and Centre for Public Health (CPH), School of Medicine, Dentistry & Biomedical Sciences, Queen's University Belfast, Belfast, U.K.

[7]Northern Ireland Centre for Stratified Medicine, Biomedical Sciences Research Institute, School of Biomedical Sciences, Ulster University, Londonderry, U.K.

Key Words: Liver, cancer, HCV, Interferon, β-defensin 1

Running Title: DEFB1 expression in HCV infected liver / liver cancer

*Corresponding Author:

Dr. Guo-Hong Deng
Department of Infectious Diseases,
Southwest Hospital,
Third Military Medical University,
Chongqing 400038, P.R. China
Email: gh_deng@hotmail.com

Prof. Hang Fai Kwok
Faculty of Health Sciences,
University of Macau,
Avenida de Universidade, Taipa,
Macau SAR
Email: hfkwok@umac.mo

Prof. Yao Lin
College of Life Sciences,
Fujian Normal University,
Fuzhou, Fujian Province,
P.R. China
Email: yaolin@fjnu.edu.cn

Supplementary Figure 1

A

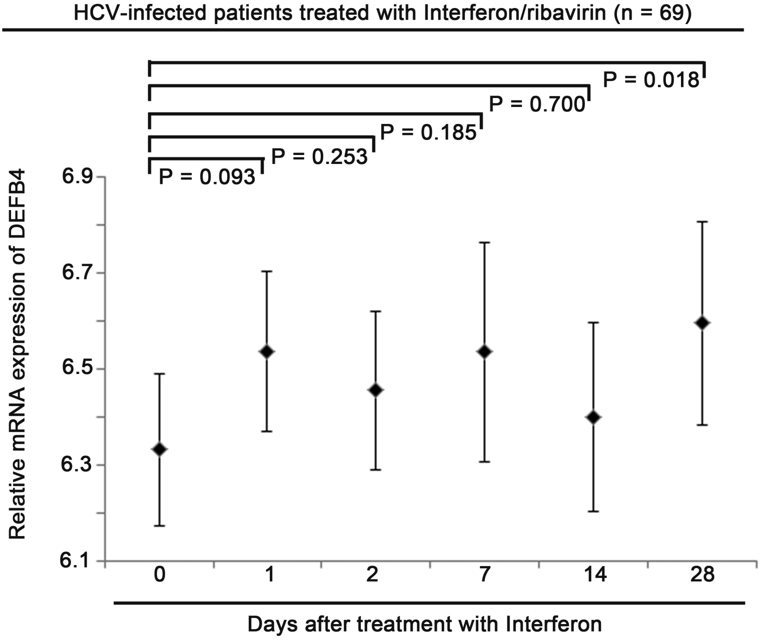

B

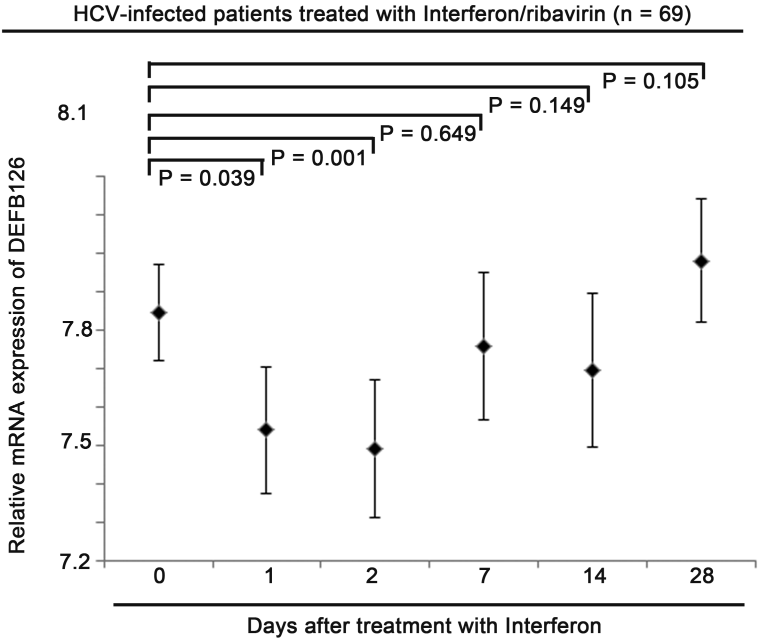

Supplementary Table 1A_GSE25907_NT

Descriptives

		N	Mean	Std. Deviation	Std. Error	95% Confidence Interval for Mean		Minimum	Maximum	p-value
						Lower Bound	Upper Bound			
DEFB1	non-tumor	243	1.991297	.6866105	.0440461	1.904534	2.078059	.5910	5.1061	.000
	tumor	268	1.206227	1.1366203	.0694301	1.069527	1.342928	.0198	5.5982	
	Total	511	1.579558	1.0266775	.0454175	1.490329	1.668786	.0198	5.5982	
DEFB110_1	non-tumor	243	.017806	.0264386	.0016960	.014465	.021147	.0115	.4243	.232
	tumor	268	.015749	.0042867	.0002619	.015234	.016265	.0101	.0598	
	Total	511	.016727	.0185030	.0008185	.015119	.018335	.0101	.4243	
DEFB110_2	non-tumor	243	.033880	.0065092	.0004176	.033058	.034703	.0193	.0679	.938
	tumor	268	.033831	.0079296	.0004844	.032877	.034784	.0199	.0768	
	Total	511	.033854	.0072818	.0003221	.033221	.034487	.0193	.0768	
DEFB112	non-tumor	243	.028359	.0051997	.0003336	.027702	.029016	.0186	.0495	.058
	tumor	268	.029351	.0065924	.0004027	.028558	.030144	.0206	.0929	
	Total	511	.028879	.0059856	.0002648	.028359	.029400	.0186	.0929	
DEFB113	non-tumor	243	.038376	.0342295	.0021958	.034051	.042701	.0185	.4364	.362
	tumor	268	.034927	.0503108	.0030732	.028876	.040978	.0164	.8026	
	Total	511	.036567	.0434060	.0019202	.032795	.040339	.0164	.8026	
DEFB114	non-tumor	243	.049490	.1034666	.0066374	.036416	.062564	.0110	1.2137	.466
	tumor	268	.041135	.1526080	.0093220	.022781	.059489	.0087	2.0956	
	Total	511	.045108	.1314908	.0058168	.033680	.056536	.0087	2.0956	
DEFB115	non-tumor	243	.017015	.0068355	.0004385	.016152	.017879	.0105	.0884	.786
	tumor	268	.016875	.0044329	.0002708	.016342	.017409	.0078	.0582	
	Total	511	.016942	.0056977	.0002521	.016447	.017437	.0078	.0884	
DEFB116	non-tumor	243	.025990	.0055227	.0003543	.025292	.026688	.0144	.0583	.262
	tumor	268	.025427	.0057912	.0003538	.024731	.026124	.0163	.0600	
	Total	511	.025695	.0056665	.0002507	.025202	.026187	.0144	.0600	
DEFB117	non-tumor	243	.013078	.0067109	.0004305	.012230	.013926	.0084	.1003	.470
	tumor	268	.012731	.0034345	.0002098	.012318	.013144	.0075	.0498	
	Total	511	.012896	.0052513	.0002323	.012440	.013353	.0075	.1003	
DEFB118	non-tumor	243	.018166	.0039785	.0002552	.017664	.018669	.0112	.0390	.765
	tumor	268	.018057	.0042841	.0002617	.017542	.018572	.0111	.0482	
	Total	511	.018109	.0041379	.0001831	.017749	.018469	.0111	.0482	
DEFB121	non-tumor	243	.022767	.0048768	.0003128	.022151	.023384	.0147	.0614	.027
	tumor	268	.021761	.0053811	.0003287	.021114	.022408	.0136	.0772	
	Total	511	.022240	.0051670	.0002286	.021791	.022689	.0136	.0772	
DEFB122	non-tumor	243	.026647	.0211056	.0013539	.023980	.029314	.0157	.2520	.978
	tumor	268	.026712	.0313439	.0019146	.022942	.030481	.0118	.4294	
	Total	511	.026681	.0269389	.0011917	.024340	.029022	.0118	.4294	
DEFB123	non-tumor	243	.041340	.0096609	.0006197	.040120	.042561	.0227	.0995	.032
	tumor	268	.039185	.0129370	.0007903	.037629	.040741	.0203	.1608	
	Total	511	.040210	.0115356	.0005103	.039207	.041213	.0203	.1608	
DEFB124	non-tumor	243	.093046	.0273810	.0017565	.089586	.096506	.0532	.3116	.043
	tumor	268	.088520	.0225608	.0013781	.085807	.091233	.0512	.2348	
	Total	511	.090672	.0250467	.0011080	.088495	.092849	.0512	.3116	
DEFB125	non-tumor	243	.019573	.0047749	.0003063	.018970	.020176	.0110	.0489	.335
	tumor	268	.019080	.0066956	.0004090	.018274	.019885	.0130	.0962	
	Total	511	.019314	.0058609	.0002593	.018805	.019824	.0110	.0962	
DEFB126	non-tumor	243	.011214	.0022242	.0001427	.010933	.011495	.0074	.0233	.022
	tumor	268	.013441	.0156106	.0009536	.011563	.015318	.0074	.2384	
	Total	511	.012382	.0114527	.0005066	.011387	.013377	.0074	.2384	
DEFB127	non-tumor	243	.026186	.0086638	.0005558	.025091	.027281	.0165	.1065	.252
	tumor	268	.025232	.0101483	.0006199	.024011	.026452	.0169	.1491	
	Total	511	.025686	.0094743	.0004191	.024862	.026509	.0165	.1491	
DEFB128	non-tumor	243	.031946	.0083657	.0005367	.030888	.033003	.0177	.0808	.131
	tumor	268	.030828	.0083267	.0005086	.029826	.031829	.0150	.0724	
	Total	511	.031359	.0083558	.0003696	.030633	.032086	.0150	.0808	
DEFB129	non-tumor	243	.014220	.0050301	.0003227	.013584	.014855	.0092	.0631	.017
	tumor	268	.013308	.0032728	.0001999	.012915	.013702	.0079	.0372	
	Total	511	.013742	.0042215	.0001867	.013375	.014109	.0079	.0631	
DEFB130	non-tumor	243	.028042	.0161550	.0010363	.026000	.030083	.0154	.1835	.028
	tumor	268	.025407	.0096816	.0005914	.024243	.026572	.0150	.1024	
	Total	511	.026660	.0132154	.0005846	.025512	.027809	.0150	.1835	
DEFB131	non-tumor	243	.025258	.0070201	.0004503	.024371	.026145	.0137	.0724	.000
	tumor	268	.029920	.0171687	.0010487	.027855	.031985	.0155	.2571	
	Total	511	.027703	.0135327	.0005987	.026527	.028879	.0137	.2571	
DEFB132	non-tumor	243	.058379	.1103061	.0070761	.044441	.072318	.0107	1.0670	.000
	tumor	268	.228846	.7511327	.0458827	.138508	.319184	.0094	10.0748	
	Total	511	.147783	.5553473	.0245671	.099517	.196048	.0094	10.0748	
DEFB136	non-tumor	243	.028871	.0155724	.0009990	.026903	.030839	.0151	.2444	.637
	tumor	268	.028349	.0076948	.0004700	.027423	.029274	.0178	.0792	
	Total	511	.028597	.0120886	.0005348	.027546	.029648	.0151	.2444	

Supplementary Table 1B_GSE14520_NT

Descriptives

		N	Mean	Std. Deviation	Std. Error	95% Confidence Interval for Mean Lower Bound	95% Confidence Interval for Mean Upper Bound	Minimum	Maximum	P=value
DEFB1	1	220	9.0678	.91730	.06184	8.9459	9.1897	3.58	10.52	.000
	2	225	7.7737	2.51009	.16734	7.4440	8.1035	3.33	12.49	
	Total	445	8.4135	2.00331	.09497	8.2269	8.6002	3.33	12.49	
DEFB4	1	220	3.9579	.28795	.01941	3.9197	3.9962	3.41	5.30	.979
	2	225	3.9572	.30712	.02047	3.9168	3.9975	3.34	5.31	
	Total	445	3.9575	.29746	.01410	3.9298	3.9853	3.34	5.31	
DEFB126	1	220	3.3183	.20789	.01402	3.2907	3.3460	2.97	4.84	.021
	2	225	3.2763	.17241	.01149	3.2537	3.2990	2.96	4.09	
	Total	445	3.2971	.19172	.00909	3.2792	3.3150	2.96	4.84	

Supplementary Table 1C_GSE36376_NT

Descriptives

		N	Mean	Std. Deviation	Std. Error	95% Confidence Interval for Mean		Minimum	Maximum	p-value
						Lower Bound	Upper Bound			
DEFB1	1.00	193	11.3541	.70968	.05108	11.2534	11.4549	9.18	13.43	.000
	2.00	240	10.2215	2.22072	.14335	9.9391	10.5039	6.02	14.32	
	Total	433	10.7263	1.80828	.08690	10.5555	10.8971	6.02	14.32	
DEFB4	1.00	193	6.5294	.15739	.01133	6.5071	6.5518	6.14	6.99	.699
	2.00	240	6.5354	.16067	.01037	6.5149	6.5558	6.08	6.98	
	Total	433	6.5327	.15906	.00764	6.5177	6.5477	6.08	6.99	
DEFB32	1.00	193	6.5562	.14268	.01027	6.5359	6.5764	6.13	6.89	.010
	2.00	240	6.5989	.20084	.01296	6.5734	6.6244	6.13	7.59	
	Total	433	6.5799	.17837	.00857	6.5630	6.5967	6.13	7.59	
DEFB32_2	1.00	193	5.5394	.13906	.01001	5.5196	5.5591	5.16	5.89	.564
	2.00	240	5.5241	.38048	.02456	5.4757	5.5724	0.00	5.93	
	Total	433	5.5309	.29790	.01432	5.5027	5.5590	0.00	5.93	
DEFB103A	1.00	193	5.8304	.13945	.01004	5.8106	5.8502	5.47	6.50	.745
	2.00	240	5.8349	.14766	.00953	5.8161	5.8537	5.48	6.42	
	Total	433	5.8329	.14391	.00692	5.8193	5.8465	5.47	6.50	
DEFB103B	1.00	193	6.5059	.18960	.01365	6.4790	6.5328	5.90	7.13	.040
	2.00	240	6.5448	.20128	.01299	6.5192	6.5704	6.05	7.72	
	Total	433	6.5274	.19689	.00946	6.5089	6.5460	5.90	7.72	
DEFB103B_2	1.00	193	6.6188	.14531	.01046	6.5981	6.6394	6.26	7.11	.346
	2.00	240	6.6333	.17450	.01126	6.6111	6.6554	6.24	7.57	
	Total	433	6.6268	.16212	.00779	6.6115	6.6421	6.24	7.57	
DEFB104A	1.00	193	6.4758	.16022	.01153	6.4530	6.4985	5.87	7.11	.391
	2.00	240	6.4620	.17105	.01104	6.4403	6.4838	5.86	6.88	
	Total	433	6.4682	.16626	.00799	6.4525	6.4839	5.86	7.11	
DEFB104B	1.00	193	6.7096	.15149	.01090	6.6881	6.7311	6.36	7.53	.010
	2.00	240	6.7510	.18019	.01163	6.7281	6.7740	6.25	7.37	
	Total	433	6.7326	.16908	.00813	6.7166	6.7486	6.25	7.53	
DEFB105A	1.00	193	5.5942	.12018	.00865	5.5771	5.6112	5.26	5.93	.047
	2.00	240	5.6171	.11731	.00757	5.6022	5.6320	5.28	6.09	
	Total	433	5.6069	.11901	.00572	5.5956	5.6181	5.26	6.09	
DEFB105B	1.00	193	6.1949	.16097	.01159	6.1721	6.2178	5.76	6.64	.939
	2.00	240	6.1962	.16789	.01084	6.1748	6.2175	5.66	6.76	
	Total	433	6.1956	.16465	.00791	6.1801	6.2112	5.66	6.76	
DEFB106A	1.00	193	6.5523	.14480	.01042	6.5317	6.5728	6.17	6.95	.651
	2.00	240	6.5592	.17033	.01099	6.5375	6.5808	6.05	7.19	
	Total	433	6.5561	.15931	.00766	6.5410	6.5711	6.05	7.19	
DEFB106B	1.00	193	6.5892	.15181	.01093	6.5676	6.6107	6.08	7.12	.830
	2.00	240	6.5860	.15704	.01014	6.5660	6.6060	5.97	7.15	
	Total	433	6.5874	.15456	.00743	6.5728	6.6020	5.97	7.15	
DEFB107A	1.00	193	5.8520	.11653	.00839	5.8355	5.8685	5.58	6.26	.366
	2.00	240	5.8624	.12235	.00790	5.8469	5.8780	5.53	6.26	
	Total	433	5.8578	.11977	.00576	5.8465	5.8691	5.53	6.26	
DEFB107B	1.00	193	6.2159	.14585	.01050	6.1952	6.2366	5.81	6.56	.135
	2.00	240	6.1947	.14627	.00944	6.1761	6.2133	5.81	6.67	
	Total	433	6.2042	.14630	.00703	6.1904	6.2180	5.81	6.67	
DEFB108B	1.00	193	5.6821	.11859	.00854	5.6653	5.6989	5.38	5.94	.519
	2.00	240	5.6899	.13200	.00852	5.6731	5.7067	5.20	6.03	
	Total	433	5.6864	.12612	.00606	5.6745	5.6983	5.20	6.03	
DEFB108B_2	1.00	193	6.4180	.15572	.01121	6.3959	6.4401	6.11	7.01	.954
	2.00	240	6.4188	.15200	.00981	6.3995	6.4382	5.88	6.78	
	Total	433	6.4184	.15349	.00738	6.4039	6.4329	5.88	7.01	
DEFB109	1.00	193	5.5730	.11501	.00828	5.5567	5.5893	5.25	5.86	.908
	2.00	240	5.5743	.11862	.00766	5.5592	5.5894	5.25	5.91	
	Total	433	5.5737	.11689	.00562	5.5627	5.5848	5.25	5.91	
DEFB109P1	1.00	193	6.2212	.13107	.00943	6.2026	6.2398	5.86	6.65	.267
	2.00	240	6.2066	.14314	.00924	6.1884	6.2248	5.83	6.59	
	Total	433	6.2131	.13793	.00663	6.2001	6.2261	5.83	6.65	
DEFB110	1.00	193	6.3367	.16160	.01163	6.3138	6.3597	5.91	7.01	.096
	2.00	240	6.3105	.16385	.01058	6.2897	6.3314	5.83	6.84	
	Total	433	6.3222	.16318	.00784	6.3068	6.3376	5.83	7.01	
DEFB110_2	1.00	193	5.7190	.11756	.00846	5.7023	5.7357	5.39	6.05	.777
	2.00	240	5.7157	.12003	.00775	5.7005	5.7310	5.41	6.01	
	Total	433	5.7172	.11881	.00571	5.7060	5.7284	5.39	6.05	
DEFB112	1.00	193	6.5535	.14272	.01027	6.5332	6.5737	6.09	6.99	.434
	2.00	240	6.5424	.14960	.00966	6.5234	6.5614	6.19	7.03	
	Total	433	6.5473	.14651	.00704	6.5335	6.5612	6.09	7.03	
DEFB113	1.00	193	6.4046	.16478	.01186	6.3812	6.4280	5.89	6.83	.928
	2.00	240	6.4061	.16644	.01074	6.3849	6.4272	5.91	7.02	

	Total	433	6.4054	.16551	.00795	6.3898	6.4210	5.89	7.02	
DEFB114	1.00	193	6.6720	.18673	.01344	6.6455	6.6985	5.91	7.51	.902
	2.00	240	6.6698	.18425	.01189	6.6464	6.6932	6.19	7.20	
	Total	433	6.6708	.18514	.00890	6.6533	6.6883	5.91	7.51	
DEFB114_2	1.00	193	5.5979	.42089	.03030	5.5381	5.6576	0.00	5.98	.242
	2.00	240	5.6346	.12416	.00801	5.6188	5.6504	5.28	5.95	
	Total	433	5.6182	.29597	.01422	5.5903	5.6462	0.00	5.98	
DEFB115	1.00	193	6.1901	.13121	.00944	6.1715	6.2087	5.77	6.52	.271
	2.00	240	6.1758	.13776	.00889	6.1583	6.1933	5.78	6.68	
	Total	433	6.1822	.13491	.00648	6.1694	6.1949	5.77	6.68	
DEBF116	1.00	193	6.3371	.14487	.01043	6.3165	6.3576	5.90	6.86	.352
	2.00	240	6.3500	.14114	.00911	6.3320	6.3679	5.86	6.66	
	Total	433	6.3442	.14280	.00686	6.3307	6.3577	5.86	6.86	
DEFB117	1.00	193	6.2736	.13913	.01001	6.2539	6.2934	5.91	6.66	.288
	2.00	240	6.2589	.14760	.00953	6.2402	6.2777	5.86	6.67	
	Total	433	6.2655	.14391	.00692	6.2519	6.2791	5.86	6.67	
DEFB118	1.00	193	5.8103	.12171	.00876	5.7930	5.8276	5.50	6.23	.161
	2.00	240	5.7932	.13167	.00850	5.7764	5.8099	5.38	6.17	
	Total	433	5.8008	.12747	.00613	5.7888	5.8128	5.38	6.23	
DEFB119	1.00	193	5.7840	.12707	.00915	5.7660	5.8021	5.37	6.09	.774
	2.00	240	5.7804	.13204	.00852	5.7636	5.7972	5.32	6.16	
	Total	433	5.7820	.12971	.00623	5.7698	5.7943	5.32	6.16	
DEFB119_2	1.00	193	6.2980	.16142	.01162	6.2751	6.3209	5.75	6.73	.799
	2.00	240	6.2939	.17504	.01130	6.2716	6.3161	5.85	6.86	
	Total	433	6.2957	.16893	.00812	6.2798	6.3117	5.75	6.86	
DEFB121	1.00	193	5.6831	.15511	.01116	5.6611	5.7051	5.28	6.18	.516
	2.00	240	5.6733	.15521	.01002	5.6536	5.6931	5.22	6.15	
	Total	433	5.6777	.15506	.00745	5.6630	5.6923	5.22	6.18	
DEFB122	1.00	193	6.6649	.13976	.01006	6.6450	6.6847	6.26	7.15	.274
	2.00	240	6.6487	.16777	.01083	6.6273	6.6700	6.24	7.44	
	Total	433	6.6559	.15594	.00749	6.6412	6.6706	6.24	7.44	
DEFB123	1.00	193	7.0719	.28107	.02023	7.0320	7.1118	6.56	8.50	.386
	2.00	240	7.0973	.32646	.02107	7.0558	7.1388	6.44	8.55	
	Total	433	7.0860	.30698	.01475	7.0570	7.1150	6.44	8.55	
DEFB124	1.00	193	5.7967	.13905	.01001	5.7769	5.8164	5.44	6.17	.437
	2.00	240	5.8075	.14981	.00967	5.7884	5.8265	5.29	6.26	
	Total	433	5.8027	.14505	.00697	5.7890	5.8164	5.29	6.26	
DEFB125	1.00	193	6.4549	.16103	.01159	6.4321	6.4778	5.90	6.93	.009
	2.00	240	6.4119	.18034	.01164	6.3890	6.4349	5.97	7.44	
	Total	433	6.4311	.17313	.00832	6.4147	6.4475	5.90	7.44	
DEFB126	1.00	193	5.6671	.11516	.00829	5.6508	5.6835	5.32	5.99	.001
	2.00	240	5.7085	.14836	.00958	5.6896	5.7273	5.22	6.51	
	Total	433	5.6901	.13600	.00654	5.6772	5.7029	5.22	6.51	
DEFB127	1.00	193	6.6805	.15581	.01122	6.6584	6.7026	6.15	7.20	.021
	2.00	240	6.7154	.15672	.01012	6.6955	6.7353	6.38	7.37	
	Total	433	6.6998	.15710	.00755	6.6850	6.7147	6.15	7.37	
DEFB128	1.00	193	6.6704	.14144	.01018	6.6503	6.6904	6.32	7.24	.265
	2.00	240	6.6865	.15919	.01028	6.6663	6.7067	6.22	7.35	
	Total	433	6.6793	.15158	.00728	6.6650	6.6936	6.22	7.35	
DEFB129	1.00	193	6.1818	.15779	.01136	6.1594	6.2043	5.77	6.57	.421
	2.00	240	6.1689	.17559	.01133	6.1466	6.1913	5.57	6.61	
	Total	433	6.1747	.16782	.00807	6.1588	6.1905	5.57	6.61	
DEFB130	1.00	193	6.1914	.16153	.01163	6.1685	6.2143	5.66	6.69	.500
	2.00	240	6.2018	.15527	.01002	6.1820	6.2215	5.73	6.67	
	Total	433	6.1971	.15799	.00759	6.1822	6.2121	5.66	6.69	
DEFB131	1.00	193	6.1965	.14742	.01061	6.1756	6.2174	5.81	6.61	.140
	2.00	240	6.1741	.16812	.01085	6.1527	6.1955	5.75	6.75	
	Total	433	6.1841	.15944	.00766	6.1690	6.1991	5.75	6.75	
DEFB131_2	1.00	193	6.6761	.15908	.01145	6.6535	6.6987	6.27	7.17	.566
	2.00	240	6.6853	.17547	.01133	6.6630	6.7076	6.31	7.41	
	Total	433	6.6812	.16823	.00808	6.6653	6.6971	6.27	7.41	
DEFB132	1.00	193	6.2068	.15661	.01127	6.1846	6.2291	5.76	6.64	.085
	2.00	240	6.2335	.16442	.01061	6.2126	6.2544	5.75	6.65	
	Total	433	6.2216	.16135	.00775	6.2064	6.2369	5.75	6.65	
DEFB137	1.00	193	5.7343	.13033	.00938	5.7158	5.7528	5.33	6.20	.852
	2.00	240	5.7366	.12558	.00811	5.7207	5.7526	5.40	6.10	
	Total	433	5.7356	.12758	.00613	5.7235	5.7476	5.33	6.20	

Supplementary Table 2. DEFB1-n32.sig.sscmap

Candidate drugs that may have the potential to reverse the gene signature you specified.

Compoud	Replicate	pvalue	zscore	significance_mark	Perturb_stability
BRD-A70514680__articaine	63	1.95E-08	-5.494871326	1	1
BRD-K86434416__selegiline	137	3.50E-08	-5.391003287	1	1
BRD-A96255180__ribavirin	46	1.73E-07	-5.095969393	1	1
BRD-A92651262__nalbuphine	65	2.13E-06	-4.598654468	1	1
BRD-K04548931__pidorubicine	85	4.35E-06	-4.447022097	1	1
BRD-A67981824__cefotaxime	45	5.72E-06	-4.387925423	1	1
BRD-A97674275__ranolazine	109	5.74E-06	-4.387414085	1	1
BRD-K69650333__idarubicin	29	6.90E-06	-4.347178303	1	1
BRD-K11469942__fluticasone	11	9.12E-06	-4.285410815	1	1
BRD-A73909368__dactinomycin	46	1.06E-05	-4.251242287	1	1
BRD-K43389675__daunorubicin	115	1.34E-05	-4.199156101	1	1
BRD-A42571354__cetirizine	121	1.46E-05	-4.179500531	1	1
BRD-K52237148__cefazolin	11	1.47E-05	-4.177893748	1	1
BRD-K14705039__sulfathiazole	10	1.57E-05	-4.163641796	1	1
BRD-A97454584__nadolol	60	1.72E-05	-4.142352894	1	1
BRD-K27218697__rimexolone	11	1.83E-05	-4.127660545	1	1
BRD-K59163477__cisapride	63	1.86E-05	-4.123821315	1	1
BRD-K18618618__cimetidine	72	1.97E-05	-4.111091469	1	1
BRD-A76941896__doxorubicin	54	4.67E-05	-3.907086594	1	1
BRD-K17210248__daunorubicin	52	4.94E-05	-3.893428353	1	1
BRD-K86873305__piperacillin	43	5.20E-05	-3.881058186	1	1
BRD-K70401845__erlotinib	214	5.95E-05	-3.848270706	1	1
BRD-K21680192__mitoxantrone	343	7.20E-05	-3.801135853	1	1
BRD-K82236179__dicoumarol	12	7.26E-05	-3.799219437	1	1
BRD-A82396632__miconazole	10	1.01E-04	-3.716837282	1	1
BRD-K55696337__topotecan	31	1.03E-04	-3.712465039	1	1
BRD-A01643550__prednisolone	80	1.22E-04	-3.669340293	1	1
BRD-K91966436__daunorubicin	9	1.24E-04	-3.665202998	1	1
BRD-K81029756__diltiazem	72	1.29E-04	-3.653796004	1	1
BRD-A68009927__daunorubicin	190	1.37E-04	-3.638463836	1	1
BRD-U73308409__vemurafenib	144	2.62E-04	-3.467860524	1	1
